# Young Transgender and Gender Nonconforming Persons Seeking Endocrine Care in the University Hospital Nancy: Lessons Learned and Challenges

**DOI:** 10.1111/psrh.70048

**Published:** 2025-12-01

**Authors:** Eva Feigerlova

**Affiliations:** ^1^ Centre Hospitalier Universitaire and Medical Faculty, Université de Lorraine Nancy France; ^2^ INSERM UMR_ S 1256—NGERE, Université de Lorraine Nancy France

## Abstract

**Introduction:**

Over the last decade at the University Hospital of Nancy in Lorraine, France, we have observed an increasing number of people under 35 years old who receive consultation for gender incongruence, from an average of 7 new patients per year in 2002–2013 to an average of 27 per year in 2014–2017.

**Methods:**

We conducted a mixed‐methods study, including a retrospective quantitative analysis of medical records of youths who sought care for gender incongruence from 2004 to 2020, and a qualitative analysis of in‐depth interviews with 11 patients identified through the medical records.

**Results:**

The study included 235 participants (135 assigned female at birth, 100 assigned male at birth). Transgender men were younger than transgender women: mean age 20 (1.6, standard deviation, [SD]) years vs. 22.7 (4.3 SD) years; *p* = 0.01 at first referral. We observed no difference in age at the initiation of gender‐affirming hormonal treatments. More than half of our participants chronologically situated their first questioning about their gender identity in the prepubertal period. Their life experiences revealed a lack of transgender representation in society, discomfort with the treatments offered, difficulties in becoming aware of and disclosing their gender identity, and the importance of peer/community support.

**Conclusion:**

The present study provides insights into the growing population of transgender and gender nonconforming people receiving care in the University Hospital of Nancy which has coincided with the evolution of the national legal framework. Our results identify several priorities for transgender youth who are receiving gender‐affirming care. Further research outside hospital networks appears warranted.

## 1 Introduction

Transgender, gender nonbinary, and gender nonconforming people have a gender identity that does not match the societal characteristics of the sex they were assigned at birth. Gender nonbinary or nonconforming people may identify with genders that are neither male nor female or a blend of both. The International Classification of Diseases (11th ed.) recently replaced the diagnostic category “transsexualism” with “gender incongruence of adolescence and adulthood,” [1] a welcome change for those who experience gender dysphoria, a clinically significant distress or impairment related to a strong desire to be of another gender [2]. Although not all transgender or gender nonconforming people experience dysphoria [2], they may seek gender‐affirming care to align their gender expression with their true gender [3, 4] to avoid discrimination [5]. The legal and social consequences of receiving/not receiving gender‐affirming care may vary depending on the local environment [6]. Transgender and gender nonconforming people in Global North countries may be confronted with hostile social environments, including rejection from family and friends, and no legal and/or social recognition of their gender identity [7]. Indeed, a recent report on legal gender recognition in Europe indicated a lack of public awareness about transgender and gender nonconforming people [8].

### 1.1 The French Legal Context

In France, the binary sex designation of citizens is a fundamental aspect of their civil status and is established by examining external genitalia at birth [9]. Until the 1990s, it was nearly impossible to change sex designation in civil status documents (e.g., birth certificates, marriage certificates), unless there was proof of an error in sex assignment at birth. Consequently, a person could attempt to modify their gender expression through clothing and/or medical treatments, but there was no guarantee that people could obtain a new civil identity. In response to ongoing discrimination and invasion of privacy concerns related to the incongruence between sexual organs and gender expression among transgender people, legislators modified Article 61–5 of the French civil code, such that a sex designation change to a citizen's civil status no longer required any justification [10]. This amendment provided a legal procedure for adults and emancipated minors in the *Tribunaux de grande instance* (Courts of First Instance) to modify sex in civil status documents, requiring only that applicants demonstrate they will “benefit from having the preferred gender,” partially de‐medicalizing the procedure. These simplified administrative procedures and societal changes have led to an increase in medical consultations for gender‐affirming hormonal treatments.

### 1.2 Healthcare Services for Transgender People in France

The French health care system offers universal coverage for all citizens, regardless of their age or economic situation. People seeking gender‐affirming care have access to both public and private hospitals and benefit from health services including preventive care and “services related to sex change” or “sex reassignment surgery” pursuant to the health insurance scheme known as *affection de longue durée (ALD)* (long‐term illness). International guidance documents for providing gender‐affirming care have evolved over the last two decades [11]. In 2009, the French National Authority for Health suggested that medical care should be provided by multidisciplinary medical teams [12]. A 2022 report requested by the Ministry of Social Affairs and Health recommended the active participation of transgender persons in medical care [13]. The French National Authority for Health proposed national standards of care [14].

### 1.3 Focus and Aims of the Study

We completed this study in Nancy, the largest metropolitan city in Lorraine, a department in the Grand Est administrative region in northeastern France. Lorraine is the only region in France to share its borders with three countries: Belgium, Luxembourg, and Germany. It includes rural and urban areas and thus people seeking care in this region come from different socio‐cultural environments.

Over the last decade, the Department of Endocrinology of the University Hospital of Nancy has observed an increase in referrals of young people (under 35 years of age) for gender‐affirming care, from an average of 7 subjects per year between 2002 and 2013, to an average of 27 subjects per year between 2014 and 2017 [15]. These numbers are consistent with statistics reported by the National Health Insurance Fund. Nearly 9000 people benefitted from the ALD insurance scheme for *transidentité* (“transidentity”) between 2013 and 2020, a tenfold increase from 2002 to 2013. Nearly 70% of the beneficiaries were between 18 and 35 years old [11]. This increase in demand for gender‐affirming care is causing health policymakers to consider guidelines for healthcare providers, particularly for younger popualtions [16]. It has also prompted questions about the difference between gender identity and sex assigned at birth and how to best identify the key moments or milestones in the journey of someone who is questioning her/his/their gender identity. In this study, we set out to identify the characteristics of young transgender and gender nonconforming people seeking care in our facility and explore the care experiences and concerns of transitioning transgender youth.

## 2 Materials and Methods

We conducted a mixed‐methods study composed of two parts. First, we conducted a retrospective review of medical records from patients who sought care for gender incongruence in the University Hospital of Nancy, one of the regional referral centers for transgender and nonconforming persons in France. Eligible participants: (1) received a diagnosis of gender incongruence; (2) were 18 years of age or older at the time of inclusion in the study; (3) were under 35 years of age when they were first referred to the endocrinology department; and (4) attended a follow‐up appointment in our department. Second, we conducted semi‐structured, in‐depth interviews with a subset of patients included in the medical record analysis following the Good Reporting of a Mixed Methods Study criteria [17].

### 2.1 Quantitative Analysis

#### 2.1.1 Screening Setting

Initial screening is part of the standard of care for all patients in the department. Screenings include a complete examination of patients' past medical history and measurement of their hormonal, metabolic, and cardiovascular status. Then patients undergo a specific endocrinological evaluation, including a physician‐led structured interview to gather the following data: self‐reported age at first questioning of their gender identity, education level, professional background, reaction of the family regarding the announcement of gender transition, and neurological or psychiatric conditions present prior to the onset of gender incongruence. Our multidisciplinary team discusses all patient records before initiating hormonal treatment, ensuring that any underlying psychiatric disorders are stabilized, and conducts routine psychiatric follow‐ups throughout the hormonal transition. The team regularly updates patients' clinical records throughout their care.

#### 2.1.2 Data Extraction for Analysis

EF, the principal investigator and an endocrinologist and cisgender woman, conducted a retrospective analysis of eligible patients' medical records who sought gender‐affirming care between January 2004 and August 2020. She extracted data from both the initial screening and the physician interview. We classified patients' self‐declared gender identities as binary (i.e., transgender man or transgender woman), non‐binary (i.e., a gender identity that involves being both a man and a woman, or is fluid, in between, or outside of the binary), or unknown (i.e., missing data in the medical record). We categorized self‐reported age at patients' first questioning of their gender identity according to patients' relation to mean age at puberty (i.e., before, during, or after puberty) [18, 19].

### 2.2 In‐Depth Interviews

In 2020–2021 we conducted in‐depth interviews with a subset of patients identified from the medical records. We excluded patients who were unable to speak French or unable to participate in a 2‐h interview. EF recruited participants by inviting eligible patients during a regular follow‐up clinical visit. Because of the difficulties associated with organizing face‐to‐face sessions during the COVID‐19 pandemic, we ultimately recruited 11 participants. MPJ, a sociologist, anthropologist, and cisgender woman, conducted the interviews with the assistance of eight postgraduate students (three cisgender women, five cisgender men, and one non‐binary transgender person) from the Master's program in Sociology at Lorraine University who were enrolled in a visual anthropology course. All participants agreed to this format and signed a consent form to that effect. The interviewers and interviewees did not meet or know each other before the interview. The interviewees chose the place (e.g., park, café, home, in‐person, or by video) and the time of the interview, and only participants and researchers were present. We audio recorded (*n* = 1) and/or video recorded (*n* = 10) all interviews with consent from participants. The duration of the interviews varied between one and 2 h. Interviewers made notes during and after the interview and we subsequently transcribed interviews in French; we offered participants the opportunity to read these transcripts and provide feedback and corrections. MPJ transcribed and coded eight interviews and the postgraduate students transcribed and coded three. MPJ analyzed all interviews. The study follows the *consolidated criteria for reporting qualitative research* [20].

### 2.3 Ethics

The study is registered at ClinicalTrials.gov and the French National Commission for Data Protection and Liberties. Before inclusion, all participants received written and oral information regarding the study objectives, voluntary participation, and assurance of confidentiality. The patients who participated in the in‐depth interviews signed the consent form before participating. We anonymized all data extracted from medical records and we masked data from the interviews with pseudonyms.

### 2.4 Data Analysis

#### 2.4.1 Quantitative Data Analysis

We express all quantitative data as mean and standard deviation (SD), including age at first referral, age at initiation of hormonal treatment, and age at gender‐affirming surgery. We carried out comparisons between groups using the Mann–Whitney U test. Quantitative data related to proportions are expressed as percentages, including the proportion of patients receiving gender‐affirming hormonal treatment, the proportion of patients receiving gender‐affirming surgery, self‐reported age at first questioning of gender identity (i.e., before puberty, during puberty, after puberty, unknown), self‐identified gender identity (i.e., binary, non‐binary, unknown), reaction of the family to the announcement of gender transition (i.e., against, indifference, support, unknown), highest level of education attained, and the presence of a neurological or psychiatric condition prior to the onset of gender incongruence according to disease categories present in the medical records. We performed statistical analyses using Microsoft Excel and the publicly available platform BiostaTGV (Pierre Louis UMR Institute S 1136; 2017). We consider a *P* value of < 0.05 as statistically significant.

#### 2.4.2 Qualitative Data Analysis

We analyzed transcripts using a grounded theory approach [21, 22] based on domains of inquiry that included: (1) becoming aware of their transgender identity; (2) revealing their gender identity to relatives and others; and (3) interacting with the medical department, family, and peer support. We formulated all research questions prior to recruiting interviewees. We began to analyze the data after the first few interviews and we moved back and forth between recruitment, data collection, and analysis.

## 3 Results

### 3.1 Participant Characteristics

**FIGURE 1:**
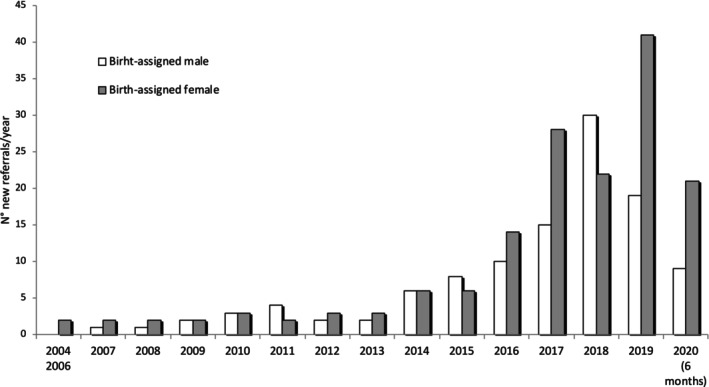
Evolution of referrals of young transgender and gender nonconforming subjects < 35 years to endocrine care in the Gender Identity Centre of the University Hospital of Nancy between 2004 and 2020 (6‐month data are provided for 2020).

We identified a total of 256 adults eligible to participate in the study. Seven patients did not give their consent and data were missing for 14 patients. In all, 235 transgender and gender nonconforming persons (135 assigned female at birth (AFAB) and 100 assigned male at birth (AMAB)) participated in the study, including the 11 participants we interviewed. Before 2007, there were fewer than 5 new referrals to endocrine care per year and this number remained relatively stable until 2013. Since 2014, we noticed a marked increase in the number of first consultations in endocrinology, with a growing proportion of people AFAB (Figure 1). A greater proportion of participants AFAB self‐reported first questioning their gender identity before puberty (71.1%) than those AMAB (56%) (Table 1). At the first referral to endocrine care, participants AFAB were significantly younger than participants AMAB: mean age 20.6 (1.6 SD) years vs. 22.7 (4.3 SD) years (*p* = 0.01), and a majority self‐identified as a binary gender (72.7% AFAB and 76% AMAB). Most participants had no neurologic or psychiatric condition prior to the onset of gender incongruence. There were no differences between participants AFAB and participants AMAB regarding education and family environment, which suggests similar social contexts in both groups. Thirty percent of our patients did not have a high school diploma, 7.6% had a Bachelor's degree, and 7.2% had a Master's degree.

**TABLE 1:** Sociodemographic characteristics of the participants at first referral to endocrine care (*N* = 235).

	Assigned female at birth (*N* = 135)	Assigned male at birth (*N* = 100)	*p*
Age at first referral^a^	20.6 (1.6)	22.7 (4.3)	0.01
Gender affirming hormonal treatment^b^	109 (80%)	82 (82%)	NS
Age at initiation of hormonal treatment^a^	22.1 (3.7)	24 (4.9)	NS
Gender affirming surgery^b^	35 (25.9%)	19 (19%)	NS
Age at gender affirming surgery^a^	24.8 (4.1)	26.7 (4.4)	NS
Self‐reported age at first questioning on one's gender identity^b^			
Before puberty	96 (71.1%)	56 (56%)	NS
During puberty	31 (23%)	31 (31%)	
After puberty	2 (1.5%)	4 (4%)	
Missing data	6 (4.4%)	9 (9%)	
Education^b^			
Master's degree	8 (5.9%)	9 (9%)	NS
Bachelor's degree	12 (8.8%)	6 (6%)	
High school graduation	46 (34.2%)	28 (28%)	
Less than high school	38 (28.1%)	34 (34%)	
Missing data	31 (23%)	23 (23%)	
Self‐identified gender identity^b^			
Binary	98 (72.7%)	76 (76%)	NS
Non‐binary	36 (26.6%)	23 (23%)	
Missing data	1 (0.7%)	1 (1%)	
Reaction of the family to gender transition^b^			
Against	8 (6%)	13 (13%)	NS
Indifference	8 (6%)	5 (5%)	
Support	77 (57%)	47 (47%)	
Missing data	42 (31%)	35 (35%)	
Neurologic or psychiatric disability prior to the onset of gender incongruence			
None	94 (70%)	65 (65%)	NS
Anxiety	11 (8.1%)	9 (9%)	
Depression	10 (7.4%)	9 (9%)	
Eating disorder	9 (6.6%)	7 (7%)	
Unspecified psychiatric disorder	3 (2.2%)	2 (2%)	
Trauma^c^	2 (1.4%)	1 (1%)	
Suicidal self‐injury	3 (2.2%)	3 (3%)	
Non‐suicidal self‐harming injury	2 (1.4%)	2 (2%)	
Schizophrenia	1 (0.7%)	2 (2%)	
Neurodevelopmental disorder	0	0	

^a^ Data expressed as mean (SD).

^b^ Data expressed as numbers and percentages (%).

^c^ Social, family, gender/sex‐related, psychiatric, other.

### 3.2 Lived Experiences

Interviewees' narratives were structured along several axes, beginning with a list of elements that, from childhood to adulthood, brought evidence of the troubles and the solutions put in place over time to remedy them. Participants reported becoming aware of their gender identity at varying ages, and expressed the importance of peer support. However, the 11 interviews allowed no generalization of the lived experiences of the patients, though they did help to describe the diversity of the experiences. The six central themes that emerged from the interviews are as follows:

○ Becoming aware of transgender identity: chaotic, difficult
○ Revealing transgender identity to third parties
○ Transition experiences: discovery and acceptance of the new body
○ Absence of an identification model for transgender persons in society
○ Discomfort at the proposed solutions
○ Importance of support by peers and by the community.

These experiences can be summarized into three categories: the discovery of transgender identity, the importance of peer support, and the personal experiences related to social and/or physical transition.

#### 3.2.1 Discovery of a Transgender Identity

All participants described concrete experiences that led them to question their gender identity, and highlighted several important moments that can be structured along two axes. The first axis concerns interviewees' experiences of various social situations that led them to question their gender identity and make different decisions. Simon (man, 33 years old) described:

I used to tell them: I'm a boy, I'm not a girl, I don't have my period anyway, I don't have breasts, I have a lot of hair everywhere. There was a problem… And my whole family actually told me that it was going to come when I grow up, that I was going to become more feminine, that it was adolescence, but that I was a girl. […] But all my childhood friends said to me “she is not our girlfriend, she's our guy”. […] I started to work and then at the age of 18 I joined the army for 5 years; I was a commando in the air force. As a woman, I wanted to prove that women were not worth less than men. While I had already chosen my new first name.

On the other hand, Martin (man, 30 years old) experienced awareness of his gender identity through searching for information on the Internet:

So, I would see trans people but, like most people I think, I believed that they were men who had become women. In fact, I never saw the inverse situation. It is not shown, or you can't see it, I don't know. And so, I did not know that it was possible. So, thanks for the Internet, actually! (laughter) I started talking about it with my relatives when I understood what happened and found solutions on my own.

These social experiences can be expressed at various ages. Interviewees who questioned their gender identity as children explained feeling that they were born in the wrong body, and often recounted anecdotes about clothing. For example, Paul (man, 29 years old) said, “When I had to wear dresses I would cry, and I would wear pants because I can't stand dresses. I have three sisters and I don't do anything like them”. Simon (man, 33 years old) explained: I realized that I am a boy when I was three. My mom wanted to dress me as a girl, and it was a nightmare. I wanted to play basketball like my brother, and I started playing soccer. Or Alice (woman, 24 years old), who told us about the questions she asked as a child about clothing: “for marriages, why do I have to wear a tie that chokes my neck while the girls can all look super pretty?”

During puberty, participants indicated having trouble with two experiences: bodily changes and heteronormativity. Whether participants experienced puberty early (10 years) or late (14 years), the men we interviewed expressed difficulty dealing with bodily changes during puberty, like the development of buttocks, hips, and more pronounced breasts, even before their first period. Participants who desired a masculine gender expression would hide their bodies in large clothes. Alain (man, 20 years old) explained, “as my brother is only one year older than me, I would borrow his clothes”. Some men explained that this discomfort coincided with the moment they became aware of their attraction to girls, as Bryan (man, 19 years old) said, “I would look at them on the street, not that I wanted to look like them, but I saw that they were pretty. I realized that I was sexually attracted to them”. Several interviewees told us about a phase in which they thought they were gay, before realizing that they were transgender. This refers to various approaches of considering gender and its relations to sex and sexuality, while making a difference between these three dimensions [23].

Participants also said that issues related to their gender identity interfered with their studies, causing them to fall behind, sometimes leading them to abandon their education.

The second axis concerns disclosure of transgender identity to close friends, family, school or work, medical personnel, or psychologists, or to a member of a nonprofit organization, which may be voluntary or coerced. Celine (woman, 28 years old) recounts a complicated journey to assert her female identity as the only child of five assigned male at birth:

I had long periods where I would tune out things I didn't want to hear about it. I would buy clothes on the Internet and, sometimes, I put everything in a bag in the back of the closet. And then two months later, I had to get everything out again at all costs. It was, it was impossible to live with it, but also to live without it… So, it was a very complicated path, with ups and downs, and a lot of questioning. Between puberty and, let's say, early adulthood, where, well, I had to take charge of myself.

Throughout the interview, Celine described how her gender identity was incomprehensible and unacceptable to her relatives because of the privileges she enjoyed as the only child assigned male at birth. On the other hand, Cyril (man, 25 years old) used social networks to begin to assume his male identity, presenting himself as a boy online and showing a photo of his friend's brother. His mother gradually understood the situation and decided to support him in his gender transition. In addition, two interviewees preferred to reveal their gender identity to their siblings rather than their parents. They explained that age proximity made their experiences more understandable to their siblings.

All participants described coming out as both delicate and frightening, within the family and among friends. Leon (male, age 19 years) reported frequently getting into trouble as a teenager, before putting into words the gender he identified with: “I did a lot of stupid stuff in high school. We emptied fire extinguishers, turned the staff room upside down, were disrespectful to adults, I was always getting expelled, and I even got in trouble with the law. Nothing serious, but it was constant”. Then, he went on to explain that since he named his transgender feelings, his behavior improved. In contrast, Lucas (male, age 18 years) described:

After the summer, I was certain. A Friday in September, I told to my two best girlfriends from high school. Throughout the weekend, they texted me to explain it was all nonsense. That I was just doing it to get noticed. I thought to myself, if this is what it's like with them, how is it going to be with my parents or in society? I was so disappointed. On Sunday evening, I took 11 pills. It was in the hospital that I talked to the psychiatrist. He was there when I had to tell my parents. Since then, my mother is very present, she comes with me to the appointments.

#### 3.2.2 Peer Support

All 11 interviewees described a lack of social models that would help them understand their feelings. The interviewees emphasized the importance of the trans community in helping them put words to their experiences and think about their future. For example, Ctibor (man, 28 years old) explained, “She [his partner] saw me watching videos, she saw me asking myself questions, and then one day I just told her, this is where I want to go, and I want to start”. Ctibor related how sharing his own experiences and feelings in online communities helped his self‐identification. Alois (male, 19 years old) explained, “We talk about stages, possibilities, feelings. We also often exchange addresses of ‘safe’ doctors, because some doctors can have very violent reactions to what we are going through”. For example, Bryan (male, 19 years old) said:

I knew S. at school. She—he was much older than me. He was very quiet for a while and then one day he posted ‘Here I am S. Now I'm a boy’. I immediately wrote ‘I wish you lots of strength’ but I wasn't surprised. He was the first person I've seen come out. If it was easy for him, then why wouldn't it be easy for me? He's been a guide for me on Instagram and has allowed me to meet a lot of people.

#### 3.2.3 Personal Experiences Related to Social and/or Physical Transition

Finally, the third category explores the personal experiences of social and/or physical transition. The interviewees described administrative and medical steps such as changing their first name, the effects of hormone treatments, the discovery and appropriation of the new body and new types of sexuality, and the consequences on their social relationships. Celine (female, 28 years old) explained that it was difficult to make her desire to transition acceptable to family, friends, and her workplace. Sometimes, participants described the workplace as more understanding than family, or vice versa. This is the case for Celine who works in public infrastructure, which is an extremely masculine environment.

Regarding physical changes, the interviewees explained that gender affirmation surgery is not necessary for a successful gender transition, but may be for others. Several participants supported this statement, citing the pain and risks involved, like necrosis or infection. For example, Martine (woman, 24 years old) explains: “What I have in my pants is my own business and not a problem for my partner.” However, for others, like Leonard (man, 28 years old), the operation is essential to feel and be identified as a man.

## 4 Discussion

Our data show that the age of becoming aware of one's gender identity is variable, and that the school environment often plays a central role in the questioning and expression of one's gender identity. It is of note that a case of suicide of a transgender high school student in 2020, together with an increasing number of students with gender identity questions, led the Ministry of National Education to publish a circular letter in 2021 “for better consideration of gender identity issues in schools.*”* [24] This corresponds with the literature which shows that gender expression is particularly important in communities of trans youths [25, 26].

Our data further show a strong overrepresentation of transgender men. Since 2016, the population of transgender men seeking gender‐affirming care in our service outnumbers that of transgender women. This contrasts with the work of Guillot and Beaubatie who have studied the invisibilization of transgender men in medical, psychological, and sociological scientific inquiries since 2003, where transgender men are hardly present [27]. The authors posit that, both socially and in the media, becoming a man is considered a normal desire, since it means joining the dominant group, whereas the transition of someone assigned male at birth is seen as illogical, and therefore worth investigating. Interestingly, nine of the 11 transgender people we interviewed self‐identify as men. This potentially indicates their desire to be visible and indicates a new direction in gender representation among youth.

The third notable point in our results is the contrast between participants' desire for gender affirmation through hormonal treatments, instead of through surgery. Note that both testosterone and estrogen hormone treatments are associated with an increased risk of adverse effects [28]. The interviews show how young transgender men don't require strict synergy between their sexual organs, gender, and sexuality to feel affirmed. Our quantitative data indicate that 77% of individuals who self‐identify as a binary gender did not undergo reassignment bottom surgery. This phenomenon is described in the literature, and does not contradict transgender people's experiences of being affirmed in their gender identity [25, 26, 29].

There are several other explanations for these findings which involve the context of gender‐affirming care in France. Firstly, the quantitative part summarizes two decades of follow‐up and thus minimizes recent changes, whereas the qualitative part focuses on the past 2 years, which are marked by new societal understandings of gender, sex, and sexuality. Secondly, French law required gender confirmation surgery to change civil status documents before 2010, and binarity remains the principle that underlies law and society. The procedure of modifying one's first name is now carried out by an officer invested with public authority, and no longer by a judge, and the modification of sex designation in civil status documents has been de‐medicalized, but remains a judicial decision based on proof that changing gender will benefit the applicant.

Our observations further show that the self‐identification process is variable, in terms of gender, class, and age [23]. However, in the context of the French legal and medical systems, transgender persons still have to demonstrate a need for medically assisted transition to obtain financial support for treatments and/or surgeries. Transgender identity thus remains marked by marginalization [30], exceptionality, and the diversity of individual paths can be grouped under our six major themes depicted through the interviewees' lived experiences. Michel Foucault analyzed the close link between medicine and the judiciary in his course for the Collège de France in 1974–1975 [31]. He indicates it as a source of knowledge and a power of normalization. One of the ways to address marginalization, transform individual experiences into collective knowledge [32], and influence unsatisfactory legal and medical proposals, is to compare experiences. As other research has shown [33, 34, 35], our interviewees revealed the importance of peer support through social networks and non‐governmental organizations. The trans community is therefore not only a place to share information and testimonies, but also a place of socialization, substituting other forms of socialization, such as family or school. However, data from the literature show that sometimes there are conflicts within trans communities due to class and gender relations. Indeed, gender has a strong influence on the way social areas are understood and on the activities that are considered acceptable within them [36]. Life experiences cannot be understood without contextualizing them against the loaded backdrop of a binary gender construction in French society [10]. As Butler mentioned 30 years ago [37], transgender identity raises questions about gender and other issues related to gender, which should be considered as national and even international social issues.

### 4.1 Limitations

This study is retrospective and some data are missing. We did not include data from the very first referrals between 1989 and 2002 due to the paucity of information available in the medical records. We supplemented the data during follow‐up visits to obtain the most accurate clinical information on our participants. Also, 8% of participants were not included because of missing data or the absence of consent to participate. Our qualitative analyses are exploratory and we did not achieve data saturation. As it stands, our data do not allow us to evaluate any difference between those who seek care at the university hospital and those who do not.

## 5 Conclusions

The major findings are a strong overrepresentation of transmen, the emergence of gender expressions beyond traditional binary models, and a marked increase in the trans population. Lorraine, the study site, is the only French region to share its borders with three other countries, that it is a combination of rural and urban areas, and that people who request care in our hospital department therefore come from different socio‐cultural backgrounds. However, these social issues also concern the healthcare system, educational institutions, and family environments. Our study calls for other research studies to be carried out outside hospital networks.

## Funding

The author has nothing to report.

## Conflicts of Interest

The author declares no conflicts of interest.
